# Magnetic tuning of liquid crystal dielectric metasurfaces

**DOI:** 10.1515/nanoph-2022-0101

**Published:** 2022-06-01

**Authors:** Yana V. Izdebskaya, Ziwei Yang, Mingkai Liu, Duk-Yong Choi, Andrei Komar, Dragomir N. Neshev, Ilya V. Shadrivov

**Affiliations:** ARC Centre of Excellence for Transformative Meta-Optical Systems (TMOS), Research School of Physics, The Australian National University, Canberra, ACT 2601, Australia

**Keywords:** dielectric resonators, liquid crystals, optical anisotropy, optical metasurfaces, tunable metasurfaces

## Abstract

Dielectric metasurfaces hold an exceptional potential for the next generation of tunable optical systems that find applications in sensing, ranging, and imaging. Here, we introduce and demonstrate magnetic field tuning of dielectric metasurfaces infiltrated with liquid crystals. To illustrate this concept, we show how the reorientation of liquid crystal induced by the magnetic field changes the spectrum of the resonant dielectric metasurface. This new magnetic-field tuning approach offers significant advantages over other liquid crystal tuning methods since it does not require pre-alignment or the fabrication of structured electrodes, which are both challenging when dealing with metasurfaces. Furthermore, there are no strict limitations on the thickness of liquid crystal cells. Importantly, our approach allows for gradual tuning of the resonances by changing the magnetic-field orientation and, thereby, shows good promise for highly tunable optical metadevices.

## 1 Introduction

Dielectric metasurfaces provide a powerful platform for flat optical devices due to their unique ability to shape electromagnetic waves [1–5]. Advances in design and fabrication of such metasurfaces have led to creation of several ultrathin optical metadevices, including flat lenses, holograms, beam converters, and deflectors. To date, most functional dielectric metasurfaces are based on static designs, defined through geometrical parameters, such as shape and size of nanoparticles, as well as their layout. It is crucial to enable dynamic tunability of the metasurfaces in order to achieve their practical functionality. As a result, substantial effort has been applied to realize reconfigurable metasurfaces. There are several approaches to making reconfigurable metasurfaces [6], for example the geometry of the metasurfaces can be dynamically adjusted, e.g. by stretching a metasurface made on a stretchable substrate [7–9], or by tuning optical properties of nanoresonators or their surroundings [10–15]. In the latter case, functional materials with optical properties sensitive to external stimuli such as temperature or applied voltage have to be integrated into the metasurface architecture. One of the examples of such materials that allow achieving tuning of metasurfaces is liquid crystal. Nematic liquid crystals (NLCs) are particularly attractive for tunable metasurfaces owing to their large birefringence and technological advances.

Up to date, it was demonstrated that the NLC-based dielectric metasurface properties can be controlled by external stimuli, including temperature [16, 17] and voltage [18–20]. Thermal tuning is achieved when the NLC above a certain temperature threshold becomes isotropic, while electric tuning is observed when the orientation of NLC molecules, and thus the optical axis of NLCs can be controlled by an external electric field. Moreover, recently it was demonstrated that it is possible to tune the transmission properties of metasurfaces by combined application of thermal and electrical stimuli [21, 22]. While all these methods can achieve tuning of the optical response of metasurfaces, they have strong geometrical limitations due to the requiring fixed in-plane NLC molecular pre-alignment. Indeed, the initial NLC orientation is typically planar, defined by a pre-alignment layer that is produced by mechanically rubbing [18, 19] or photo-alignment methods [20]. This pre-alignment of the NLC, however, cannot be changed once the NLC cell is assembled. In addition, while the conventional NLCs pre-alignment is done by using two substrates between which the NLC is placed, in the case of metamaterials, at least one of the substrates contains a metasurface and cannot be easily used with a pre-alignment layer. The initial in-plane orientation of liquid crystals also unavoidably leads to strong dependency on the polarization of light due to the birefringence in the bulk of the NLC. In addition, the continuous tunability is quite difficult to realize [23, 24]. Typically, two-electrode NLC cell offers “on–off” performance because in all intermediate cases, when the applied electric field is not sufficiently strong, the position of the NLC director is not exactly defined.

In our work, for the first time to our knowledge, we demonstrate a novel type of dynamic control of the spectral response of NLC-infiltrated dielectric metasurfaces that is not limited by the pre-alignment or electrode geometry. Our approach uses an *external magnetic field* that induces reorientation of the anisotropic NLC in any direction. As an example, we demonstrate that by rotation of the applied magnetic field, we are able to tune the metasurface resonances gradually and demonstrate a maximum dynamic tuning range for resonances of 37 nm and 42% absolute transmission change at infrared wavelengths around 1500 nm. Without the limitations imposed by NLC boundary conditions, the magnetic tuning mechanism paves the way for new types of tunable metadevices, including spatial light modulators and optical limiters. Due to its universal nature, the method can be also used in other frequency ranges, including terahertz and microwave frequencies.

## 2 Materials and methods

**Figure 1:**
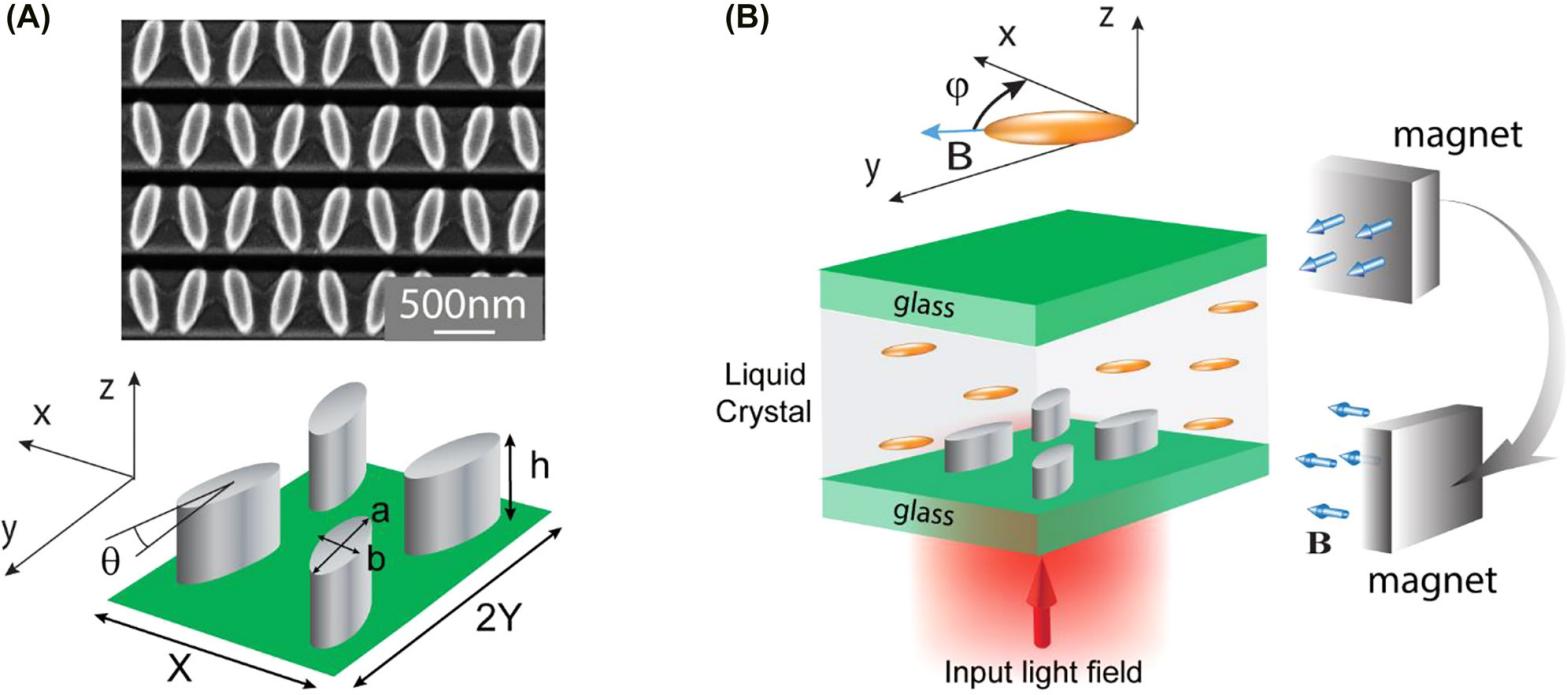
Magnetic tuning of dielectric metasurface integrated into the NLC cell: (A) Scanning electron micrograph of the fabricated metasurface. The insert below shows a sketch of dielectric metasurface that is composed of zigzag arrays of elliptical silicon cylinders with parameters *θ* = 15°, *X* = 900, *Y* = *P*_y1_ = 730, *a* = 540, *b* = 180, and *h* = 538 (units are nanometers). (B) Schematic illustration of the metasurface integrated into an NLC cell. The NLC molecules’ arrangement is defined by the direction of applied magnetic field **B** at the azimuthal angle *φ* in-plane *x*–*y*. We consider a uniform field strength and, as a result, a homogeneous distribution of *n* in the sample.

In this work, we use a dielectric metasurface made of zigzag arrays of silicon elliptical cylinders, as shown in Figure 1A [25]. We chose this structure because it demonstrates relatively high-quality resonances based on quasi-bound states in the continuum (QBIC). The fabrication of the metasurfaces was performed using electron-beam lithography on hydrogenated amorphous silicon-on-silica glass, followed by Al etch mask lift-off, reactive ion etching, and residual Al removal with a wet etch solution. Details of the sample fabrication can be found in [25]. A scanning electron micrograph image of the fabricated metasurface on a quartz substrate is shown in Figure 1 (A-top). Such metasurface design supports two high-quality factor (high-*Q*) QBIC modes [25, 26]. The modes are excited by illuminating the metasurface with an *x*-polarized light and their coupling to free space is controlled by the tilt angle of the elliptical cylinders in the *x–y* plane. In our design, the tilt angle *θ* is 15°, resulting in a quality factor of the order of 100. The two modes that we focus on correspond to the magnetic and electric QBIC resonances in the metasurface and we will call them, magnetic and electric mode, respectively.

Next, we infiltrate the metasurface with an NLC. In our experiments, we employ the NLC mixture 6CHBT [27], which feature a strong optical anisotropy along the axis of the NLC molecules in its nematic phase. It has an ordinary refractive index of *n*_o_ ∼ 1.51 and an extraordinary refractive index of *n*_e_ ∼ 1.67 at room temperature 20 °C in the infrared. Using the metasurface as the base (bottom) substrate, we fabricated an NLC cell with a 1 mm thick glass spacer. The glass spacer has a circular opening separating the metasurface from the top substrates, which is the space filled by the liquid crystal. The thickness of the NLC cell was chosen to minimize the Fabry–Perot effect due to multiple reflections. For the selected spacer thickness, these oscillations occur at a period much smaller than the spectral width of the relevant metasurface resonances. The top substrate is a ∼160 μm thick plane cover glass (without any NLC alignment layer) and it is used as the NLC cell’s output window. Finally, the infiltration of the NLC cell was performed in a vacuum to assist NLC penetration into the spaces between silicon nanocylinders.

While the general assembly scheme for the transmission-type NLC-integrated metasurface is similar to previous works [16, 18, 21], here, as a crucial advantage, we do not deploy any pre-alignment materials on the NLC boundary surfaces to define a desired orientation of the NLC. To achieve the initial orientation of NLC, we apply a sufficiently strong magnetic field that can orient the NLC molecules in any direction. By doing this, we not only avoid a reasonably complex technological step during fabrication, but we have an extra degree of freedom by aligning the NLC in an arbitrary direction, not only parallel to the interface. Such freedom can further extend the flexibility for metasurface tuning through the dynamic molecular realignment [21].

## 3 Results and discussion

We employ a white-light spectroscopy setup to measure the linear optical transmittance spectra of the metasurface integrated into the NLC cell. To ensure the collection of the transmitted light, we use a 20× microscope objective. The metasurface structure was illuminated by the incident unpolarized light, while the analyzed polarization was chosen along the *x*-axis [*φ* = 0°, see Figure 1A], parallel to the main lattice direction of the metasurface array. The light was incident from the metasurface side of the cell to ensure that the NLC layer did not affect the parameters of the incident light on the metasurface.

In order to control the orientation of the NLC molecules, we use a magnetic field, **B** [28, 29], of a permanent neodymium magnet [46 × 30 × 10 (mm)] mounted on a three-dimensional mechanical stage placed at a distance of 4 mm from the sample. Considering that the magnet size is relatively large compared to the distance between the magnet and the metasurface, the magnetic field across the sample can be assumed uniform. The magnetic field strength measured at this distance was approximately 0.4 T.

**Figure 2:**
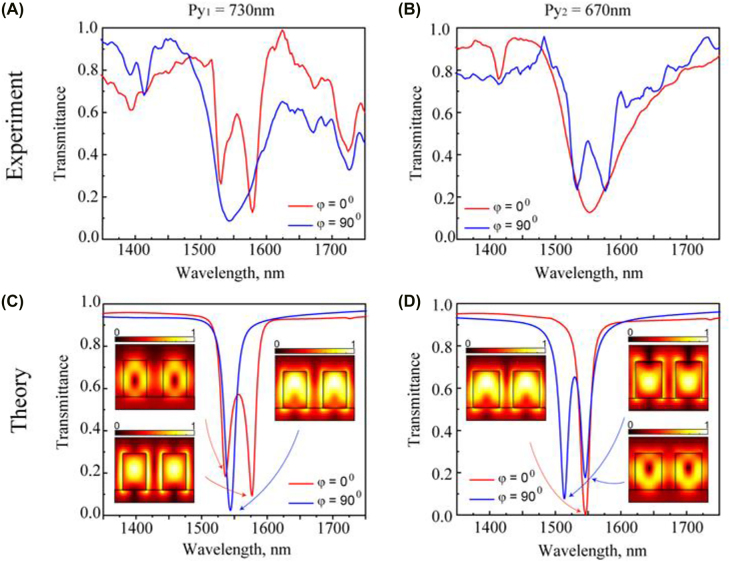
Measured normalized (A, B) and numerically calculated (C, D) transmittance spectra for two samples after NLC infiltration with the periods *P*_*y*1_ = 730 nm (A, C) and *P*_*y*2_ = 670 nm (B, D). Red lines denote the resonance positions when applied magnetic field **B** is along the *x*-axis (*φ* = 0°) while blue lines correspond to the case when **B** is along the *y*-axis (*φ* = 90°). The insets in (C, D) show the amplitude of electric field profiles of QBIC resonances.

By changing the direction of the magnetic field **B**, we are able to reorient the NLC director in *x*–*y* plane, with direction given by the azimuthal angle *φ* [Figure 1B]. We assume that the magnetic field is sufficiently strong to completely reorient the NLC molecules along vector **B** [28, 29]. In our work, we investigate two metasurfaces that have different periods in *y*-direction. Metasurface 1 has an array period *P*_*y*1_ of 730 nm, while metasurface 2 has *P*_*y*2_ = 670 nm. Figure 2A and B show the experimentally measured transmittance spectra for both metasurfaces depending on the magnetic field orientation. The measured spectra have been normalized to the transmittance through the cell without metasurfaces. First, we measured the transmittance spectra for the metasurface with *P*_*y*1_ = 730 nm [Figure 2A]. When the magnetic field **B** is applied parallel to *x*, (i.e. *φ* = 0°), we observe two resonances at *λ* ≈ 1531 nm and *λ* ≈ 1580 nm [red line in Figure 2A]. However, as the magnetic field **B** rotates by an angle *φ* = 90°, the two resonances move closer towards each other and finally, merge into a single transmission dip at *λ* ≈ 1544 nm [blue line in Figure 2A]. Remarkably, we observed the inverted dynamics for the metasurface with *P*_*y*2_ = 670 nm [Figure 2B]. In particular, in the case when *φ* = 0°, we observe the single resonance dip at *λ* ≈ 1552 nm, but when NLC alignment angle is increasing from 0° to 90°, we clearly observe splitting of major resonance dip into two resonances at *λ* ≈ 1532 nm and at *λ* ≈ 1577 nm [blue line in Figure 2B]. The maximum splitting distance is achieved for *φ* = 90°. The switching time for the transition between the states *φ* = 0° and *φ* = 90° is of up to 1 s, but we expect that stronger magnets or electromagnets will be able to induce faster reorientation with the switching time down to several ms.

Next, we compare these results with numerical calculations, and show a good agreement with the experimental spectra. To calculate the transmittance spectra of the NLC-integrated metasurface, we assume that the applied magnetic field leads to an ideal homogeneous NLC alignment along the magnetic field. The liquid crystal infiltrated zig-zag metasurface is simulated using commercial finite-difference time-domain (FDTD) software lumerical, using periodic boundary conditions along *x*- and *y*-directions and perfect matching layers (PML) along *z*-direction. The covered NLC layer is regarded as a homogeneous distribution without considering the influence of the metasurface. Figure 2C and D shows the corresponding numerical simulations for different states of NLC molecular orientation in the *x–y* plane. The spectrums of Figure 2C and D demonstrate two resonances (E-QIBC and M-QBIC) for different orientation of the NLC. More specifically, a blueshift can be observed for the E-QBIC resonance because the dominant field direction senses the refractive index decrease. On the contrary, an increased refractive index is perceived by the main field of M-QBIC, which will lead to its resonance redshift. The two resonance modes overlap to form one dip, and we note that the Huygens condition does not appear in this case. This is because the effective refractive index of the superstrate (NLC in our case) cannot match the substrate refractive index for the high Q structure [30–32]. Quantitative differences between experimental and numerical results and additional spectral features in the experimental Figure 2A are likely caused by the imperfections of the metasurface fabrication. These include roughness of the surface, variation from the initial shape and size, as well as slight symmetry breaking resulting from a slightly different tilt of the left and right ellipse (see Figure 1A). The latter symmetry breaking can possibly lead to the additional modes that can couple to free-space radiation in the experiment. Such modes can be analyzed by simulating the imperfect geometry matching the experiment as much as possible; however, this analysis remains outside the scope of the current work. The insets in Figure 2C and D show the amplitude of electric field structure in the *y*-plane cutting the top resonators in the middle, and they show when individual resonators have dominating electric dipole response (when the field has maximum in the middle of resonators), or magnetic dipole response, when there is a minimum of the field.

**Figure 3:**
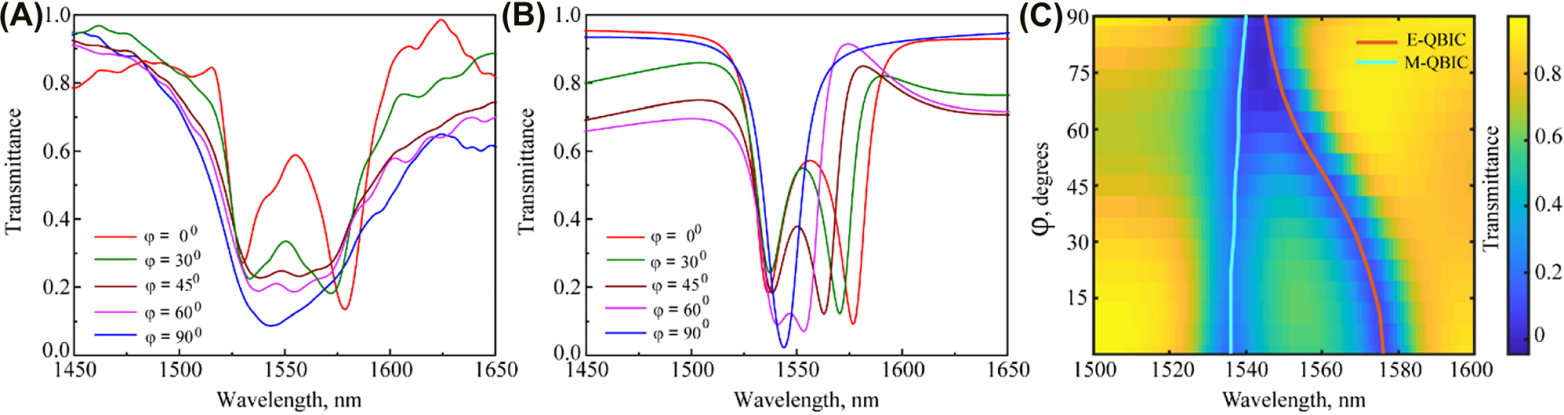
Measured (A) and corresponding numerically calculated (B) transmittance spectra for the sample with the period *P*_*y*1_ = 730 nm for different angles *φ* of the magnetic field **B** in *x*–*y* plane (*φ* = 0, 30, 60, 45, and 90°). (C) Calculated transmittance spectra of the metasurface from Figure 3B versus the systematic magnetic field orientation *φ*. Cyan and orange lines denote the spectral positions of two QBIC resonance positions.

Finally, we investigate the tuning capability of our metasurface, we record the transmittance of the sample with *P*_*y*1_ = 730 nm when we rotate the magnetic field and change the angle *φ* from 0° to 90°. Figure 3 shows our experimental and numerical results for different angles *φ*. In the case when the magnetic field orientation is along the *x*-axis (*φ* = 0°), the resonance dips are spectrally separated, and appear at about 1531 nm and 1581 nm. However, when the magnetic field rotates from *φ* = 0° to *φ* = 90° major resonance dips are gradually moving towards each other and finally overlap at around 1544 nm. We observed shifts of 13 nm for magnetic and 37 nm for the electric resonance. The measured transmission efficiency is about 42%. Overall, the measured results show a good qualitative agreement with the calculated spectra. The continuous shift of the spectral resonances offers more flexibility as compared to “on–off” switching approaches and they can be used for the design of a wider range of metasurface-based photonic systems offering versatile dynamic control of the properties of light.

In conclusion, we have realized experimentally and studied numerically, a novel type of dynamic control of dielectric metasurfaces by an external magnetic field. By rotating the magnetic field, we were able to control the molecular orientation of liquid crystal thus changing the refractive index of the medium surrounding the metasurface. We showed that the suggested magnetic tuning approach does not require pre-alignment, fabrication structured electrodes, and there is no practical limitation on the thickness of liquid crystal cells. Using dielectric silicon metasurface consisting of zigzag arrays of silicon elliptical cylinders integrated into an NLC cell, we observed how the QBIC resonances could overlap or split in transmittance at the infrared wavelengths around 1500 nm depending on the orientation of the magnetic field and array periods. We achieved experimental transmission modulations of up to 42% and a maximum dynamic tuning range for resonances of 37 nm. Furthermore, we are able to obtain a continuous tuning of spectral resonances by smooth orientation of the magnetic field. Our results entail a new approach to tuning dielectric metasurfaces for realizing tunable and switchable dielectric metadevices for wavefront manipulation. Despite our proposed method has some disadvantages compared to electrical tuning, such as the difficulty of manipulating each small “pixel” individually and the observed switching time was not fast enough, we expect that magnetic field tuning will have applications either for larger sample control or in combination with electric field, when the pixel level tuning is needed. In the latter case, magnetic field can completely replace the pre-alignment of the liquid crystal cell, which is a particularly difficult problem in case of metasurfaces. The faster switching speed and smaller device size can be achieved by using a vector magnet whose direction of the magnetic field can be dynamically changed in all three dimensions. In addition, the magnetic field penetrates easily into many dielectric materials without loss of intensity. It can be seen as an advantage in some settings, when, for example, dynamic switching of a large set of devices is required. The access to the additional way of liquid crystal tunability expands the functionality of the NLC-integrated metasurfaces due to the large refractive index change of the NLC, and our results open up several future research directions.
